# Early post-stress decrease in cardiac performance by impedance cardiography and its relationship to the severity and extent of ischemia by myocardial perfusion imaging

**DOI:** 10.1186/s12872-020-01639-2

**Published:** 2020-07-31

**Authors:** Ronen Goldkorn, Alexey Naimushin, Eli Rozen, Dov Freimark

**Affiliations:** 1 Leviev Heart Center, Sheba Medical Center, 52621 Tel Hashomer, Israel; 2 Sackler School of Medicine, Tel Aviv University, Tel Aviv, Israel

**Keywords:** Coronary artery disease, Myocardial perfusion imaging

## Abstract

**Background:**

While single photon emission computed tomography (SPECT) myocardial perfusion imaging (MPI) is a well-established noninvasive procedure for the evaluation of patients with coronary artery disease (CAD), it is unable to detect the presence of, or underestimates the extent of CAD in certain patients. We aimed to show that a bio-impedance device can detect early post-stress changes in several hemodynamic parameters, thereby serving as a potential marker for the presence of significant ischemia.

**Methods:**

Prospectively enrolled patients, referred to our Medical Center for clinically-indicated MPI, underwent testing using a Non-Invasive Cardiac System (NICaS) before and immediately after exercise. The differences between rest and stress hemodynamic parameters were compared with the severity and extent of myocardial ischemia by MPI. The study included 198 patients; mean age was 62 years, 26% were women, 54% had hypertension, and 29% diabetes mellitus. Of them, 188 patients had ≤10%, and 10 had > 10% of myocardial ischemia.

**Results:**

In the first group, there was a significantly greater increase in post-exercise stroke index, stroke work index, cardiac index and cardiac power index (19.2, 29.1, 90.5 and 107%, respectively) compared with the second group (− 2.7, 3.8, 43.7 and 53.5%, respectively), as well as a significantly greater decrease in total peripheral resistance index (− 38.7% compared with − 16.3%), with corresponding *p* values of 0.015, 0.017, 0.040, 0.016, and < 0.001, respectively.

**Conclusions:**

Our data suggest that immediate post-stress changes in several hemodynamic parameters, detected by the NICaS, can be used as an important adjunct to SPECT MPI for the early detection of myocardial ischemia.

## Background

Single photon emission computed tomography (SPECT) myocardial perfusion imaging (MPI) is a well-established noninvasive procedure for the evaluation and risk stratification of patients with coronary artery disease (CAD) [1]. However, it has been recognized that in certain patients SPECT MPI is unable to detect the presence of, or underestimates the extent of CAD. The fact that moderate to severe perfusion defects are noted in less than half of the patients with significant left main disease [2] has stimulated studies to improve the diagnostic accuracy of SPECT MPI. Such studies have analyzed post-stress parameters such as left ventricular ejection fraction [3, 4], wall motion abnormalities [5] and transient left ventricular dilation [6] with images acquired on conventional Anger cameras as long as 60 min after the stress tracer injection. Such delayed assessment may miss early ischemic stunning as a result of its transient nature.

The Non-Invasive Cardiac System (NICaS, NI Medical, Israel) is a whole-body bio-impedance device capable of measuring various hemodynamic parameters [7–11]. We hypothesized that the non-invasive detection of an early post-stress decrease in cardiac performance may serve as a potential marker for the presence of significant or extensive ischemia. We therefore aimed to test for an early post-stress change in several hemodynamic parameters by the NICaS, and its relationship to the severity and extent of myocardial ischemia in patients undergoing exercise stress MPI using a novel cadmium-zinc-telluride SPECT camera.

## Methods

### Study population

We prospectively enrolled 198 patients who were referred to the Nuclear Cardiology Center at the Sheba Medical Center, Tel Hashomer, Israel, for a clinically-indicated exercise stress MPI study. Exclusion criteria included patients with: unstable angina, decompensated heart failure, systolic blood pressure > 200 mmHg or diastolic blood pressure > 110 mmHg, uncontrolled arrhythmias, severe aortic stenosis, acute pulmonary embolism, acute myocarditis or pericarditis, acute aortic dissection, intra- and extra-cardiac shunts, hemodialysis and those aged < 18 or > 80 years of age. The study was approved by the hospital’s Institutional Review Board and all patients provided written informed consent.

### Exercise stress protocol and image acquisition sequence

Beta blockers and calcium-channel antagonists were terminated at least 24 h before testing, and nitrates at least 6 h before testing. During the pre-imaging stress-lab evaluation and procedures, standard 12 leads for ECG monitoring and leads for image gating were applied, and a venous catheter was inserted into an antecubital vein. The imaging room was equipped with an ECG monitor, emergency cart and oxygen source.

A “stress-first-rest-second” protocol was used, as previously described [12–21]. Briefly, after obtaining baseline heart rate, blood pressure, and a 12 lead ECG, a symptom-limited treadmill-exercise (Bruce protocol) was performed. At peak exercise, an IV bolus of 6-11 mCi 99mTc-sestamibi, according to a body mass index-related dose schedule, was injected. The first imaging was started at least 20 min after the IV tracer injection. The patient was placed in the supine position of the cadmium-zinc-telluride-SPECT camera (Discovery NM 530c, General Electric Healthcare, Israel). The detector was positioned to include the entire heart image, as well as to isolate the heart from extra-cardiac activity. Acquisitions utilized a 20% energy window centered around the 140 KeV peak of 99mTc-sestamibi, and a16-bin ECG-gating was performed using a 50% acceptance window. Subsequently, the patient was placed in a prone position and imaging was repeated. After at least 1.5 h, the patient returned to the lab for rest injection and acquisition using 16-31 mCi of 99mTc-sestamibi, according to a body mass index-related dose schedule. Images were reoriented into short-axis and vertical and horizontal long-axis slices using standard software (QPS/QGS, Cedars-Sinai Medical Center, Los Angeles, CA, USA). All image contours were reviewed by experienced technologists and nuclear cardiologists on a case-by-case basis and were individually adjusted if necessary [12–21].

### Automated quantification of perfusion

The QPS software computed the total perfusion deficit score by integrating the hypo-perfusion severities below normal limits in polar map coordinates [14]. Normal limit thresholds were defined as 3.0 mean absolute deviations (approximately equivalent to 2.5 standard deviations) for each polar map sample. Ischemic total perfusion deficit was calculated as an absolute difference between stress and rest total perfusion deficit [15, 16, 18], and was expressed as a percentage.

### The non-invasive cardiac system

The NICaS calculates the stroke volume by measuring impedance cardiography in a tetra-polar mode, derived from electrodes placed on one wrist and the contra-lateral ankle [7, 8]. During transmission of an electrical current through the body, resistivity to its conduction (bio-impedance) is measured. The resistivity of blood and plasma is the lowest in the body, 150 and 63 Ω/cm, respectively, while resistivity of cardiac muscle, lungs and fat is 750, 1275 and 2500 Ω/cm, respectively. Thus, when an alternating current of 32.5 kHz, 1.4 mA is delivered through the two electrodes, it is primarily distributed via the extracellular fluid and the blood, and the changes in body resistivity are therefore related to the dynamic changes of the blood and plasma volume. Therefore, the measured bio-impedance and its fluctuations over time are proportional to the stroke volume. Consequently, each systolic increase in the aortic blood volume is associated with a proportional increase in the measurable conductance of the whole body. In addition, a standard three-lead ECG connection is made for measuring the pulse rate. Patient age, gender, weight, height, hematocrit and electrolytes are entered into the NICaS when the monitoring is started and are used for the stroke volume calculation. Cardiac output is calculated by multiplying stroke volume by the heart rate.

Measurements are adjusted to the body surface area to yield stroke index (SI) and cardiac index (CI). Mean arterial pressure (MAP), calculated from standard blood pressure measurements, together with SI and CI allows the calculation of stroke work index (SWI = MAP*SI/7500.8 J/m^2^), cardiac power index (CPI=MAP*CI/451 W/m^2^) and total peripheral index (TPRI = MAP/CI*80 Dyn*Sec/cm^5^*m^2^).

This simple to operate, non-invasive technique has been validated in several studies as a reliable estimation of resting CO, compared with traditional, invasive techniques in different settings including healthy subjects, patients with heart failure and ischemia [7–11]. Evaluation of impedance was performed both before and immediately after exercise. Given that the patient needs to be motionless during the measurement to avoid motion artifacts, data acquisition was carried out about 1 min after completion of exercise. The differences between rest and stress hemodynamic parameters were compared with the severity and extent of myocardial ischemia by MPI.

### Statistics

Statistical analyses were performed using SPSS software (version 2b, IBM Corporation). All baseline variables are described as mean ± SD. Rest, stress and changes of hemodynamic parameters are described as mean and 95% confidence interval. A one-way ANOVA was used to compare differences for continuous variables. A chi-square test was used to compare differences across subgroups for categorical variables. A 2-tailed *p* < 0.05 as a cut-off was considered statistically significant. Receiver operator characteristic (ROC) curve was used to calculate sensitivity and specificity.

## Results

Patient baseline clinical characteristics and medications are presented in Table 1. Mean age was 62 (±9) years and 74% were males. A sizeable percentage of patients had CAD, with a previous myocardial infarction in 22%, previous percutaneous transluminal coronary angioplasty in 18%, and previous coronary artery bypass grafting in 5%. Notably, the characteristics of those patients with myocardial ischemia ≤10% were similar to those with myocardial ischemia > 10%, with no significant differences in any of the baseline parameters. Patient baseline (resting) hemodynamic parameters are presented in Table 2. Once again, there were no statistically significant differences between patients with myocardial ischemia ≤10% and those with myocardial ischemia > 10%.

**Table 1:** Demographics and general characteristics of all patients and of subgroups (myocardial ischemia ≤10 and > 10%)

Parameter	All patients N-198	Myocardial ischemia ≤10% - N 188	> 10% - N 10	*P* value
Myocardial ischemia (%)	2.4 ± 3.8%	1.8 ± 2.6%	14.4 ± 3.5%	> 0.001
Age (year), mean ± SD	61.8 ± 9.4	61.9 ± 9.4	60.4 ± 9.3	0.636
Male, n (%)	147 (74)	137 (72)	10 (100)	0.166
BMI (kg/m^2^), mean ± SD	27.5 ± 3.6	27.4 ± 3.5	29.5 ± 4.8	0.08
Diabetes mellitus, n (%)	58 (29.3)	54 (28.7)	3 (30)	0.923
Hypertension, n (%)	106 (53.5)	98 (52.1)	7 (70)	0.265
Smoking, n (%)	31 (15.7)	30 (16.0)	1 (10)	0.620
Dyslipidemia, n (%)	135 (68.2)	128	6 (60)	0.615
MI, n (%)	44 (22.2)	39 (20.7)	4 (40)	0.149
PTCA, n (%)	35 (17.7)	31 (16.5)	3 (30)	0.268
CABG, n (%)	10 (5.1)	9 (4.8)	1 (10)	0.894
PVD, n (%)	5 (2.5)	5 (2.7)	0	0.605
TIA, n (%)	1 (0.5)	1 (0.5)	0	0.819
Beta Blocker, n (%)	52 (26.0)	50 (26.6)	2 (20)	0.653

*BMI* Body mass index, *CABG* Coronary artery bypass grafting, *MI* Myocardial infarction, *PTCA* Percutaneous transluminal coronary angioplasty, *PVD* Peripheral vascular disease, *SD* Standard deviation, *TIA* Transient ischemic attack

**Table 2:** Baseline (rest) hemodynamic parameters of all patients and of subgroups (myocardial ischemia ≤10 and > 10%)

Parameter	All patients	Myocardial ischemia ≤10%	> 10%	*P* value
SBP (mmHg), mean ± SD	157 ± 21	157 ± 21	154 ± 24	0.729
DBP (mmHg), mean ± SD	85 ± 10	85 ± 11	87 ± 9	0.967
MAP (mmHg), mean ± SD	108 ± 12	108 ± 12	109 ± 13	0.708
HR (beats/s), mean ± SD	72 ± 12	73 ± 12	69 ± 6	0.398
SI (ml/m^2^), mean ± SD	37.2 ± 6.7	37.0	39.4 ± 6.1	0.271
SWI (J/m^2^), mean ± SD	0.50 ± 0.10	0.49	0.54	0.156
CI (l/min/m^2^), mean ± SD	2.70 ± 0.63	2.69	2.77	0.798
CPI (W/m^2^), mean ± SD	0.60 ± 0.15	0.59	0.63	0.506
TPRI (Dyn*S/cm^5^*m^2^), mean ± SD	3163 ± 905	3171	3012	0.591
GGI, mean ± SD	12.9 ± 3.5	13.0	11.3 ±	0.133

*CPI* Cardiac power index, *CI* Cardiac index, *DBP* Diastolic blood pressure, *GGI* Granov Goor index, *HR* Heart rate, *SI* Stroke index, *MAP* Mean arterial pressure, *SBP* Systolic blood pressure, *SWI* Stroke work index, *TPRI* Total peripheral resistance index

The hemodynamic changes between rest and stress in patients with myocardial ischemia ≤10% and in those with myocardial ischemia > 10% are presented in Table 3. In the first group, there was a significantly greater increase in post-exercise SI, SWI, CI and CPI (19.2, 29.1, 90.5 and 107%, respectively) compared with the second group (− 2.7, 3.8, 43.7 and 53.5%, respectively), as well as a significantly greater decrease in TPRI (− 38.7%) compared with the second group (− 16.3%), with corresponding *p* values of 0.015, 0.017, 0.040, 0.016, and < 0.001, respectively.

**Table 3:** Hemodynamic changes between rest and stress in patients with myocardial ischemia ≤10 and > 10%. Data are presented as mean (95% confidence interval)

Parameter	Myocardial Ischemia ≤10%	> 10%	*P* Value
METS	9.9 (9.5, 10.2)	11.2 (10.3, 12.0)	0.104
Time to target HR	08:38 (08:17, 08:58)	09:59 (08:56, 11:02)	0.074
Max. HR achieved	147 (145, 149)	143 (137, 149)	0.256
ΔSBP (mmHg)	12.5% (10.8, 14.2%)	6.9% (1.3, 12.4%)	0.146
ΔDBP (mmHg)	5.2% (3.4, 7.0%)	7.9% (1.3, 14.6%)	0.499
ΔMAP (mmHg)	8.4% (7.0, 9.7%)	7.8% (2.9, 12.6%)	0.764
ΔHR (beats/s)^a^	60.0% (55.4, 64.6%)	48.7% (26.7, 70.7%)	0.281
ΔSI (ml/m2)	19.2% (15.2, 23.2%)	−2.7% (−8.2, 2.7%)	0.015
ΔSWI (J/m2)	29.1% (24.4, 33.8%)	3.8% (−5.6, 13.3%)	0.017
ΔCI (l/min/m2)	90.5% (81.8, 99.2%)	43.7% (22.1, 65.3%)	0.040
ΔCPI (W/m2)	107% (97.2, 117%)	53.5% (33.7, 73.3%)	0.016
ΔTPRI	−38.7% (−41.0, −36.4%)	−16.3% (−30.7, −1.8%)	< 0.001
ΔGGI	22.2% (16.7, 27.6%)	3.8% (−3.1, 10.6%)	0.132

^a^Hemodynamic measurements were performed with an average delay of 03:30 (03:15, 03:50) minutes from end of treadmill stress. HR was recovered to 114 (111, 116) and 98 (84, 113) for the myocardial ischemia ≤10 and > 10% groups, respectively

*CI* Cardiac index, *CPI* Cardiac power index, *DBP* Diastolic blood pressure, *GGI* Granov Goor index, *HR* Heart rate, *MAP* Mean arterial pressure, *METS* Metabolic equivalents, *SBP* Systolic blood pressure, *SI* Stroke index, *SWI* Stroke work index, *TPRI* Total peripheral resistance index

**Fig. 1:**
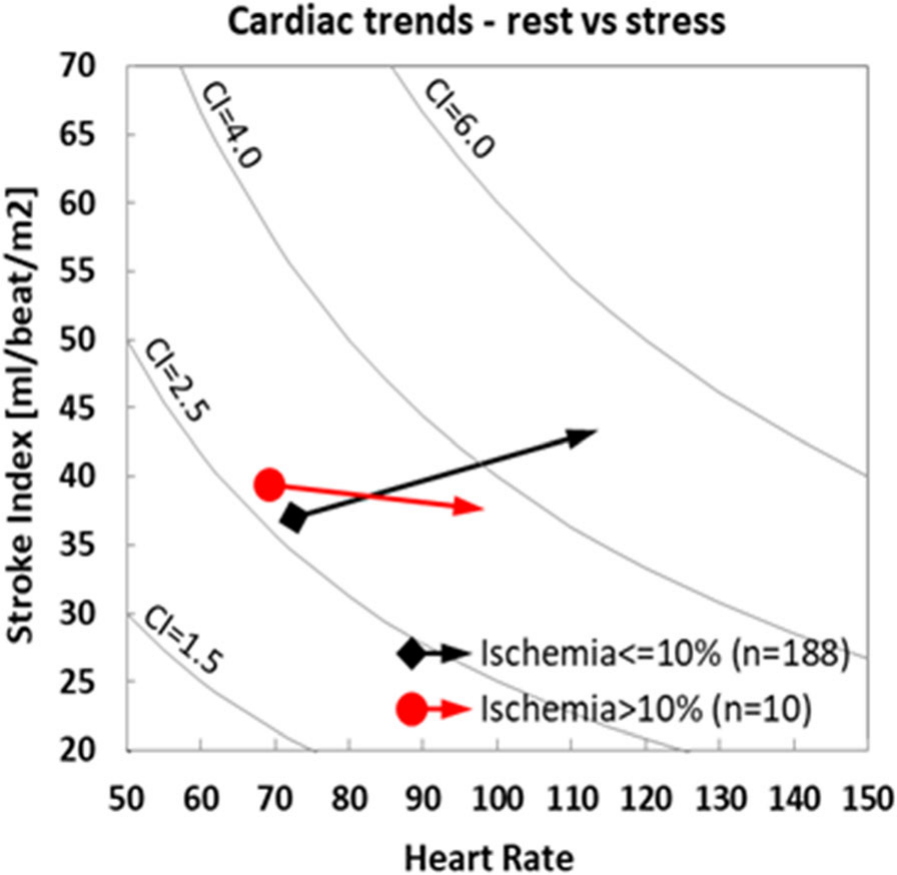
Stroke index and heart rate trends from rest to stress in patients with myocardial ischemia **≤**10% (black arrows) and in those with myocardial ischemia > 10% (red arrows). Lines of CI are also provided. CI = Cardiac index

Fig. 1 provides a visual presentation of the change in cardiac function in patients with myocardial ischemia ≤10% and in those with myocardial ischemia > 10%. As may be clearly seen, the SI in the first group increased as expected with an associated marked increase in the CI; however, the SI in the second group actually decreased and thus only a mild increase was noted in the CI, mediated only by the increased heart rate.

**Fig. 2:**
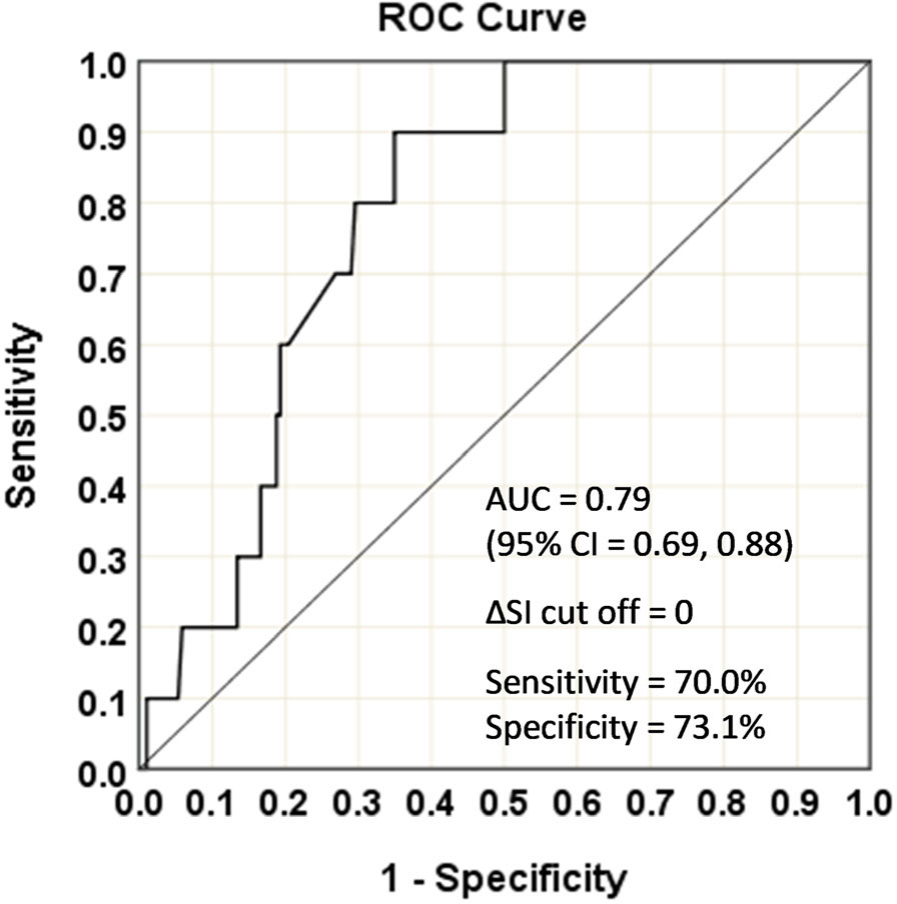
ROC curve for myocardial ischemia > 10% vs. change in SI (cut off = 0) from rest to stress. Note the AUC with 95% CI, sensitivity, and specificity. AUC = under the curve; CI = confidence interval; ROC = Receiver operator characteristic; SI = Stroke index

Fig. 2 demonstrates the ROC curve for myocardial ischemia > 10% vs. change in the SI (cut off = 0) from rest to stress. Noteworthy, are the areas under the curve with 95% confidence interval, sensitivity, and specificity. As may be seen, a change in SI of < 0 from rest to stress has 70.0% sensitivity and 73.1% specificity for detecting myocardial ischemia of > 10%.

## Discussion

While SPECT MPI is an excellent noninvasive procedure for the detection and risk stratification of patients with CAD, it may be unable to detect the presence of disease in certain patients and may underestimate its severity in others. As a result, several studies have attempted to tackle this issue using various approaches [3–6]. In this prospective study we demonstrate that the non-invasive detection of an early post-stress decrease in cardiac performance may serve as a potential marker for the presence of significant or extensive ischemia.

As already shown in multiple prior studies, the NICaS whole-body bio-impedance device is capable of reliably measuring various hemodynamic parameters [7–11]. In this study we used the NICaS to examine the hemodynamic changes between rest and stress in patients with myocardial ischemia ≤10% and in those with myocardial ischemia > 10%. As seen in Table 3, the patients with ischemia were unable to increase their SWI, CI, and CPI to the same extent as those without significant ischemia, and were unable to decrease TPRI to the same extent as those without significant ischemia.

Most notably, the SI actually decreased in those patients with myocardial ischemia > 10% (Table 3, Fig. 1). While the SI in patients without significant ischemia increased as expected with an associated marked increase in their CI, it actually decreased in those with ischemia, and therefore only a mild increase was noted in the CI, mediated only by the increased heart rate (Fig. 1). Indeed, any decrease in SI may be used to detect myocardial ischemia > 10% with 70.0% sensitivity and 73.1% specificity (Fig. 2). The novel NICaS technology consistently identifies this pattern and therefore may be an important tool in the detection of ischemia.

The current study certainly has some limitations. Beta-blocker therapy was discontinued at least 24 h prior to testing and no information was available regarding the use of other medications. In addition, our findings are applicable only to the present study population, comprising patients with an intermediate pretest probability for the presence of CAD without significant comorbidities. Moreover, the small number of patients with myocardial ischemia > 10% limits certainly impacts the strength of the data. Of note, the reproducibility of the measured hemodynamic parameters may be somewhat limited by the patients’ physical fitness and performance on the particular test day. Finally, it may also have been useful to have had echocardiographic data to look for valvular comorbidity and to have had additional validation of the stroke volume at rest. We intend to address these limitations in future work as well as to obtain correlation with true anatomy by coronary angiography.

## Conclusions

The results of the present study suggest that the immediate post-stress changes in several hemodynamic parameters as detected by the NICaS can be used as an important adjunct to the diagnostic approach for the early detection of myocardial ischemia. These findings can be used to improve risk assessment prior to a decision regarding the need to proceed with more complex and costly imaging modalities for the detection of myocardial ischemia. Moreover, future research should explore the potential use of the NICaS generated parameters as prognostic markers in the development and evolution of CAD.

## Data Availability

All data generated or analyzed during this study are included in this published article.

## Abbreviations

SPECT: Single photon emission computed tomography
MPI: Myocardial perfusion imaging
CAD: Coronary artery disease
NICaS: Non-invasive cardiac system
SI: Stroke index
CI: Cardiac index
MAP: Mean arterial pressure
CPI: Cardiac power index
TPRI: Total peripheral resistance index
SWI: Stroke work index
ROC: Receiver operator characteristic
